# Post-fatigue recovery of power, postural control and physical function in older women

**DOI:** 10.1371/journal.pone.0183483

**Published:** 2017-09-07

**Authors:** Stephen A. Foulis, Stephanie L. Jones, Richard E. van Emmerik, Jane A. Kent

**Affiliations:** Department of Kinesiology, University of Massachusetts, Amherst, Massachusetts, United States of America; University of Florida, UNITED STATES

## Abstract

Low muscle power, particularly at high velocities, has been linked to poor physical function in older adults. Any loss in muscle power following fatiguing exercise or daily activities could impact physical function and postural control until power has fully recovered. To test the overall hypothesis that a common task such as walking can result in prolonged power loss and decreased physical function and balance, 17 healthy older (66–81 years) women completed a 32-min walking test (32MWT) designed to induce neuromuscular fatigue, followed by 60min of recovery (60R). Fatigue and recovery of knee extensor muscle power (3 velocities) were quantified by dynamometry. Function was quantified by chair rise time and postural control by measures of center of pressure (COP) range (mm) and velocity (mm·s^-1^) during quiet stance. Power declined at all velocities by 8–13% 2min following the 32MWT (p≤0.02) and remained depressed by 8–26% at 60R (p≤0.04). Postural control decreased following the 32MWT, indicated by increased COP range in the anterior-posterior (AP, p<0.01) direction and a trend in the medial-lateral (ML) direction (p = 0.09), and returned to baseline by 60R (p≥0.10). COP velocity was unchanged immediately following the 32MWT, but at 60R was lower in ML (p = 0.03) and tended to be reduced in AP (p = 0.07). Changes in high-velocity power (270°·s^-1^) were associated with altered postural control (p = 0.02) and chair rise performance (p≤0.03). These results provide evidence of long-duration neuromuscular changes following fatigue in healthy older women that may place them at increased risk for functional deficits during everyday mobility tasks.

## Introduction

As humans age, they become susceptible to a number of health problems affecting muscular, postural, and physical function. Specifically, there is increased prevalence of visual, vestibular, and somatosensory dysfunction in older adults [1–3]. Age-related impairments in neuromuscular function include changes in motor unit firing behavior [1], sarcopenia [4], muscle power, and velocity-dependent muscle fatigue [5]. Ultimately, the combination of these changes can lead to changes in gait [6] and reduced postural control through increases in center of pressure sway and velocity [7]. The end result of muscle weakness and postural instability can be a reduction in physical function, i.e., the inability to perform everyday mobility tasks.

Knee extensor muscle power, the product of torque and velocity, has shown to be an important factor in maintaining physical function and mobility in older adults [8, 9]. Older adults with greater leg power have faster chair rise, stair climb and walking speeds. These associations are supported by the concept that a minimum amount of power may be necessary to perform a variety of functional tasks [10]. Above this minimum, people operate with a “functional physiological reserve,” where higher power does not affect performance of a given task. However, when power falls below this threshold, physical function declines, and eventually reaches a point where functional tasks can no longer be performed. Due to muscle weakness, older adults may operate closer to their functional reserve threshold than younger adults during everyday tasks [11]. As a result, even small changes in the amount of power they can produce, for instance as a result of fatigue, may have severe impacts on physical function.

A transient decline in power, such as that arising from muscle fatigue in response to exercise, can lower physical capacity and function [12]. While it has been shown that older adults fatigue less than young adults during isometric tasks, the opposite is true during high-velocity, dynamic tasks [5], resulting in greater power losses in the aged. It is unclear, though, how muscle power at different velocities recovers following a fatiguing exercise bout. There is some evidence for a prolonged deficit in isometric torque after an hour of recovery following a fatiguing exercise protocol [13]. In addition, Power and colleagues [14] have shown that high-speed, isotonic power remained reduced by 8% 30 min following fatiguing eccentric contractions of the ankle dorsiflexor muscles. Both studies [13, 14] attributed this incomplete recovery of torque and power to alterations in excitation-contraction coupling, which can last up to 24 hours following exercise [15].

In older adults, the functional consequences of weakness may be amplified by fatigue in some situations. Indeed, gait characteristics [16] are altered and repeated sit-to-stand times are slowed [17] following fatiguing knee extension exercise in older adults. These alterations may result in part from impaired postural control, consistent with reports of decreased center of pressure stability following fatiguing tasks in both young [18] and older adults [19]. Given that older adults are already at increased risk of falls when walking [16], any alteration in postural control could have further negative consequences. While researchers have shown functional effects from muscle fatigue induced in the laboratory by strength machines and dynamometers in older adults, an approach to fatigue that more closely reflects activities of daily living such as walking would extend our understanding of the impact of fatigue in a “real-world” setting. Furthermore, it is not known whether prolonged fatigue from this everyday activity may impact postural control or physical function.

Based on the existing literature, it is reasonable to posit that in older adults there may be deficits in postural control, physical function, and muscle power following a fatiguing walking task, and that the recovery of physical function requires adequate recovery of muscle power. Further, because it has been suggested that postural control during quiet stance is achieved, in part, through short ballistic contractions [20], lingering perturbations to the neuromuscular system in response to fatigue could be detrimental to the maintenance of balance after a fatiguing bout of exercise. Thus, the aim of this study was to evaluate the acute (i.e., 2 min post-exercise) and prolonged (60 min post-exercise) impacts of a fatiguing walking task on power, postural control, and physical function in older women. We hypothesized that in healthy older women: 1) knee extensor muscle isometric torque and dynamic power would be reduced (i.e., muscle fatigue) 2 and 60 min following a 32-min walking test (32MWT); 2) power at high velocities would be more affected by the walking task than power at low velocities; 3) physical function and postural control would be reduced 2 and 60 min following the walk; and 4) during recovery, changes in power would be associated with changes in physical function and postural stability. The results of this study have the potential to inform the design of interventions intended to mitigate loss of physical function in our aging population.

## Materials and methods

### Participants

Seventeen community-dwelling older (66–81 years) women participated in the study. All participants were relatively healthy, non-smokers free of any leg injury that could affect physical performance. No participants had a history of metabolic, neurological, cardiovascular (except for controlled hypertension), or pulmonary disease. With the exception of one participant on a low-dose beta-blocker (atenolol), whose results were well aligned with the overall group, no participant was on any medication known to affect the outcome measures of this study. All participants read and signed an informed consent document as approved by the University of Massachusetts, Amherst human subjects review board. Permission to participate was obtained from each woman’s personal physician prior to participation. Each participant made 4 visits to the Muscle Physiology Lab for consenting, habituation, and two test sessions.

All participants were sedentary to modestly active by self-report, defined as not currently training or participating in organized exercise or athletic activities. No individuals were engaged in any strength training programs. To quantify habitual physical activity, participants wore an Actigraph GT1M (Pensacola, FL) uniaxial accelerometer at the hip for seven days. Total daily activity counts, as well as daily minutes of moderate-vigorous activity (MVPA), were calculated using established thresholds for Actigraph accelerometers [21].

Following the consenting visit, all participants completed a habituation visit prior to the two testing visits. This visit occurred at least 2 days, but no more than 7 days, prior to the first testing visit. Descriptive variables evaluating anthropomorphic characteristics, functional ability, and self-reported fatigue were collected during this visit. Functional status was quantified using the Short Physical Performance Battery (SPPB; [22]), 10-s foot-tap speed [23], and 400 m walk time (10 loops of a 40 m course; [24]). Self-reported fatigue was determined using a validated questionnaire (PROMIS 7a short form; [25]). In addition, participants were familiarized with all power and functional testing measures during this visit.

Each participant then completed 2 additional visits (Muscle Power Test Session and Postural Control and Physical Function Session), which were separated by at least 48 hours. This approach allowed evaluation of the effects of the 32MWT on both muscle power and physical function with the same timing for both sets of measures. Because recovery of force following fatigue can be very rapid, combining all test measures into one session would have introduced a timing bias into the data obtained immediately following the 32MWT. The order of testing sessions was randomized and blocked across groups.

### Muscle Power Test Session

Knee extensor torque and power were measured using a Biodex System 3 dynamometer (Biodex Medical Systems, Shirley, NY), as described previously [5]. Participants were seated with the hips at 90° and a resting knee angle of 100° extension (i.e., 10° extended from the right angle). The slight extension allowed for gravity to return the leg to the resting position during dynamic contractions without requiring any muscle contraction. Torque, velocity, and position signals were collected at 2,500 Hz using a customized Matlab (Mathworks, Natick, MA) program. We used the response in the knee extensors to represent all of the muscles responsible for chair rise and balance, as knee extensor power is strongly linked to a number of functional tasks, particularly in older adults with muscle weakness [26].

Torque was recorded during knee extensor maximal voluntary isometric contractions (MVIC, Nm) at 100° lasting 3–4 s. Participants were allowed to select the leg to be tested; the same leg was used for all measures. Verbal encouragement was provided by the investigator during the MVIC, and visual feedback about torque production was provided using a lighted box. Participants performed three MVICs with 2 min of rest between contractions. Additional MVICs were performed if peak torques for two of the first three were not within 10% of each other. Peak power (W) was measured during dynamic contractions over a 70° range of motion (100°-170° of extension). Participants completed a series of three rapid contractions at each velocity. A torque-velocity curve was calculated for each participant using peak torque at 10 velocities from 30–300°· s^-1^ at 30°· s^-1^ intervals, performed in random order [5]. Each contraction series was cued by the investigator, and separated by 1 min of rest. Peak torque at each velocity was expressed relative to MVIC and fit to a second-order polynomial so that the velocity at which 75% of MVIC (V_75_) was generated could be determined. The V_75_ provided a summary variable representing each individual’s overall torque-velocity relationship [5], as well as a common relative velocity at which to perform the power measures at baseline and following the 32MWT.

### Postural Control and Physical Function Session

The effects of muscle fatigue on physical function and postural control were determined by the time to complete five rapid chair rises and postural control during 30s of quiet stance, respectively. These measures were made prior to and during recovery from the fatigue task (described below). Postural control was measured using two side-by-side force plates (AMTI, Newton, MA) to record ground reaction forces under each foot and compute measures of COP excursion. As is standard procedure for these balance measures [27], participants placed one foot on each plate parallel to each other and shoulder width apart. During the recordings, participants were instructed to place their arms across their chest and stand as still as possible with the eyes open for 30 s. Data were recorded at 120 Hz using Qualisys Track Manager (Qualysis Medical AB, Gothenburg, Sweden). Characteristics of COP excursion were quantified as the range (mm) and average velocity (mm·s^-1^) in the anterior-posterior (AP) and medial-lateral (ML) directions over each 30-s trial. Intrasession reliability for similar measures during quiet stance in older adults has been shown to be high [28].

Following the postural control test, participants completed five timed chair rises (seat height = 45 cm) with their arms folded across their chest. Chair rises were selected as a representative measure of physical function because older adults perform this task at a high percentage of their maximal strength [11], and chair rise performance has been shown to be affected by fatigue in young and older adults [17]. Chair rise time (s) was recorded as the time required to stand up completely (i.e., hips and knees completely extended) and sit back down five times as fast as possible, as described in the SPPB [22]. Time was started by cue of the investigator and stopped when the participant was seated in the chair at the end of the fifth stand-sit cycle.

### 32-Minute Walk Test (32MWT) of fatigue and recovery

Following the baseline measurements, the participant began the 32MWT protocol, which consisted of 30 min of treadmill walking followed by 2 min of overground walking. To allow accurate quantitation of fatigue-induced changes in and recovery of power, postural control measures and chair rises, participants performed the 32MWT on two days. Either power measures or chair rises and postural control measures were obtained 2 and 60 min following the walk protocol. On the first 32MWT day, treadmill speed (incline = 0°) was increased over the first 30 s until the average walking speed from the individual’s 400 m overground walk test was attained. At that point, participants were asked if they thought they could maintain that speed for 30 min. If the participant could not, treadmill speed was reduced in 0.045 m·s^-1^ increments every 10s until the participant was confident that they could complete the task. A 30 min walk time was selected for this study based on both pilot testing and the American College of Sports Medicine recommendations of 30 minutes of moderate physical activity 5 times per week for optimal health [29]. At minutes 7, 17, and 27, the grade of the treadmill was increased to 3% for 1 min in order to provide a slight challenge and to simulate a hill that the participant might encounter in everyday life. After 1 min, the grade was returned to level. To assess the subjective intensity of the walk, a rating of perceived exertion (RPE) using the Borg CR10 scale [30] was collected at minute 28. During the final 30 s, the treadmill was gradually slowed to 0.4 m·s^-1^ before stopping. Participants then stepped off the treadmill and continued walking overground for 2 min at approximately the same pace. This approach allowed us to eliminate potential motion after-effects caused by walking on a treadmill, which could negatively affect postural control [31]. This process was repeated for both fatigue tests; the same treadmill speed was used on both days.

Baseline measures of power or postural control and chair rises were collected immediately prior to the 32MWT, using the dynamometer or force platform, respectively. The first recovery measure was collected 2 min (2R) following completion of the overground portion of the 32MWT. The 2 minute delay was required to position each participant for data collection following the 32MWT. All measures were repeated following 60 min of recovery (60R). During the Muscle Power Test Session the effects of the 32MWT on muscle function were captured across a range of velocities: peak torque and power were quantified during contractions at 0°·s^-1^ (MVIC), 30°·s^-1^, V_75_, and 270°·s^-1^. Contraction order was randomized for each person and the same order was applied to all of their measurements. These four velocities were selected to allow evaluation of velocity-specific deficits in power output in response to the 32MWT on both an absolute (°·s^-1^) and subject-specific scale (V_75_). The speed for the high-velocity contractions was chosen because it was the fastest velocity all older participants could be expected to attain [32] and it is a functionally relevant velocity [33]. Fatigue was quantified as any deficit in torque (MVIC) or power production in response to the 32MWT. During the Postural Control and Physical Function Test Session, participants completed a 30-s quiet stance postural control test, followed by 5 chair rises at baseline and 2 and 60 min after the fatigue protocol. For the postural control testing, participants were positioned on the force platforms and stood quietly for 1 min prior to the start of the 30-s data collection period. On both fatigue days, participants remained seated and relaxed during recovery, with ~1 min of movement at 5, 10, 30 and 45 min of recovery. This movement consisted of the same muscle power or postural control and chair rise tasks obtained at 2 and 60 minutes of recovery (data not shown).

### Statistical analyses

All analyses were performed using SAS software (SAS Institute, Cary, NC), with significance established when p ≤ 0.05 and trends noted when p>0.05 but ≤0.10. Descriptive statistics (mean, SEM, range) were calculated to characterize the participants. To test Hypothesis 1 (as described on pg 4–5), repeated measures ANOVA (velocity, time) was used to determine the effects of 32MWT on absolute power at each time point separately. Because this analysis revealed a time x velocity interaction, post-hoc analyses using paired t tests with Bonferroni adjustments were used for independent comparisons of 2R to baseline and 60R to baseline at each velocity, to assess fatigue and recovery, respectively. To test Hypothesis 2, separate mixed-model ANOVAs were used to compare relative changes (% baseline) in torque and power across velocities at 2R and 60R. Hypothesis 3 was evaluated with 1-way repeated measures ANOVA (time), using chair-rise time and COP variables. To evaluate the relationships among changes in power and changes in chair rise and postural control measures (Hypothesis 4), linear correlation was used to calculate Pearson’s correlation coefficients (r). P values, mean ± SEM or mean and 95% (unadjusted) confidence intervals (CI) for differences from baseline are also reported, as appropriate. Effect sizes were calculated using Cohen’s d statistic. Reduced data for each individual are provided in S1 File.

## Results

### Group characteristics

Participant characteristics are summarized in Table 1. Prescription medication use included anti-hypertensives, antidepressants, anti-inflammatories, synthetic thyroid hormone, and statins. The group averaged 126 min of MVPA weekly (18 min·day^-1^). Thirteen of the 17 participants scored 12 out of 12 on the SPPB; the remaining individuals scored an 11, with one losing a point on the balance subscore and the other 3 on the chair rise test.

**Table 1:** Group characteristics.

	Mean (SEM)	Range
**Anthropometrics**		
Age (years)	70.7 (1.2)	66–81
Height (m)	1.62 (0.02)	1.49–1.71
Mass (kg)	67.4 (3.0)	52.2–98.2
BMI (kg·m^-2^)	25.8 (1.3)	17.5–36.3
**Physical Activity**		
Activity (counts·day^-1^·1000^−1^)	193 (21)	81–390
MVPA (min·day^-1^)	18.0 (4.0)	0.9–58.7
**Self-Reported Fatigue**		
PROMIS (t-score)	43.7 (1.78)	29.4–63.4
**Functional Characteristics**		
400m speed (m·s^-1^)	1.37 (0.05)	1.01–1.68
Foot taps (#·10s^-1^)	48 (10)	33–66
Chair rise, 5x (s)	8.91 (0.78)	4.34–16.12
Knee extensor MVIC (Nm)	115.2 (5.52)	80.9–149.3
V_75_ (°·s^-1^)	54.7 (4.7)	22.4–95.1

BMI: Body Mass Index; MVPA: Moderate-Vigorous Physical Activity; PROMIS: Patient Reported Outcomes Measurement Information System (7); MVIC: Maximum Voluntary Isometric Contraction; V_75_: Velocity at which 75% of MVIC torque was generated.

### 32MWT performance

Average walking speed during the 32MWT was 1.35 ± 0.05 m·s^-1^. For 13 of the 17 participants, this was the same speed as their over-ground 400 m test (Table 1). Treadmill speeds for the other four participants were reduced by up to 0.13 m·s^-1^. During the protocol, 16 of the 17 participants completed the full 30 min on the treadmill, including all 3 incline challenges. Due to fatigue in one participant, treadmill walking was stopped at the 27-min mark, after only two of the incline challenges were completed. This was followed by 2 min of overground walking and then recovery measures, as per the protocol design. Walking duration and speed were kept the same on both days for each participant. Mean RPE at the end of the 32MWT was 3.9 ± 0.4 (range: 0.5–6.0) and did not differ across days (p = 0.70), indicating the intensity of the walk was “Moderately Hard” [30] and did not differ across trials.

### Effects of the 32MWT on Muscle torque and power

**Fig 1:**
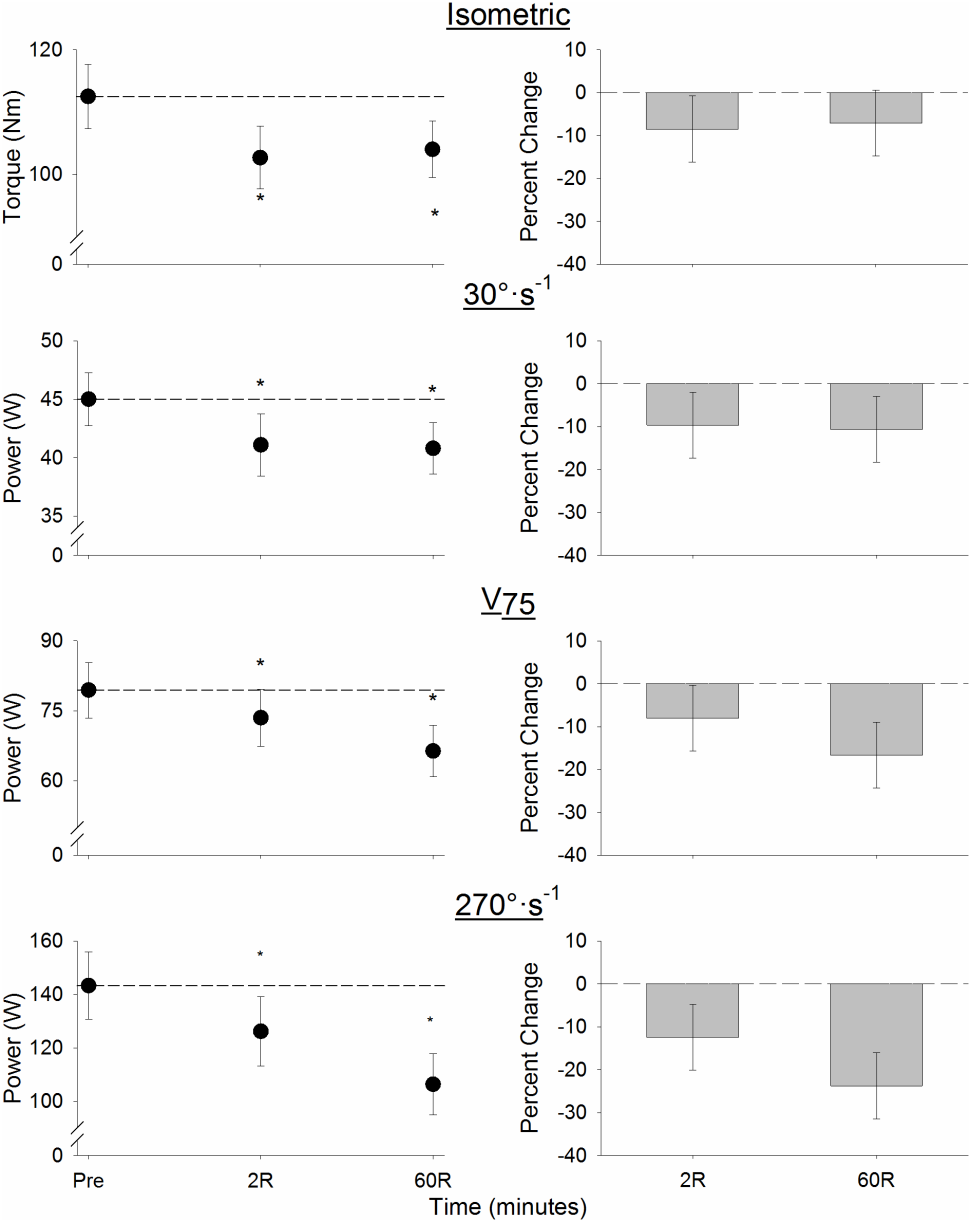
Absolute (*left*) and relative (*right*) changes in torque and power for the 4 contraction velocities. Isometric torque and power at all velocities decreased from baseline following the 32MWT (p ≤0.03, all). At 60R, the relative power deficit increased with velocity (p = 0.04), with no such differences at 2R. *Left*: mean ± SEM; *right*: mean and 95% CI for difference from baseline; dashed line indicates baseline; *p<0.05 for difference from baseline. V_75_: Velocity at which 75% of MVIC torque was generated.

Muscle torque and power fell in response to the 32MWT (Fig 1). There was a main effect of time (p<0.01) and a time x velocity interaction such that at 2R, absolute isometric torque and dynamic power had decreased from baseline at all velocities (p≤0.02; Fig 1, left panel) and remained depressed at 60R (p≤0.04). At 60R, the relative power deficit increased as velocity increased (p = 0.04; Fig 1, right panel); there were no such differences at 2R. Precise means, SD, and effect sizes are provided in Table 2.

**Table 2:** Torque, power, postural control, and physical function at baseline and during recovery.

	Baseline	2R		60R	
	Mean ± SD	Mean ± SD	ES	Mean ± SD	ES
**Torque/Power**					
0°·s^-1^ (Nm)	112.5 ± 5.1	102.7 ± 5.0*	0.87	104.0 ± 4.5*	0.83
30°·s^-1^ (W)	45.0 ± 2.3	41.1 ± 2.7*	0.71	40.8 ± 2.2*	0.64
V_75_ (W)	79.4 ± 6.0	73.5 ± 6.1*	0.77	66.4 ± 5.5*	1.47
270°·s^-1^ (W)	143.3 ± 12.6	126.1 ± 13.0*	0.83	106.3 ± 11.4*	0.96
**Postural Control**					
AP COP Range (mm)	23.1 ± 6.6	30.5 ± 11.1*	0.90	27.5 ± 9.4	0.48
ML COP Range (mm)	8.68 ± 3.1	11.7 ± 5.5	0.52	10.7 ± 4.8	0.51
AP COP Velocity (mm·s^-1^)	118.3 ± 25.0	127.9 ± 33.2	0.45	108.4 ± 22.0	0.57
ML COP Velocity (mm·s^-1^)	77.2 ± 12.1	79.8 ± 12.6	0.34	74.0 ± 9.3*	0.66
**Physical Function**					
Chair Rise (s)	8.9 ± 3.2	9.0 ± 3.2	0.15	9.1 ± 3.7	0.15

* p<0.05 for difference from baseline; ES: effect size (Cohen’s d)

### Postural control and physical function

**Fig 2:**
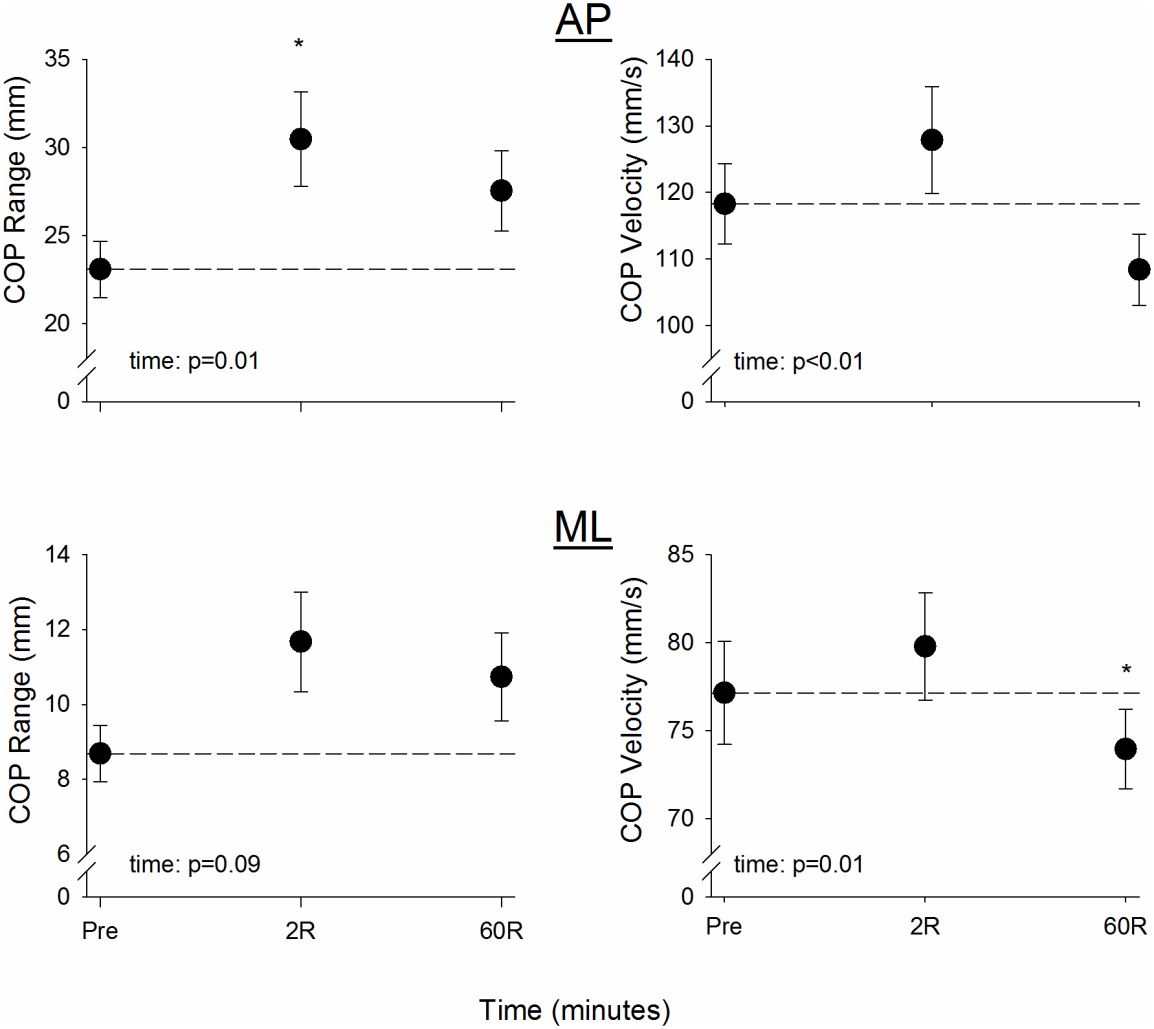
Changes in center of pressure (COP) range (*left*) and velocity (*right*) in AP (*top*) and ML (*bottom*) directions. AP COP range during quiet stance was increased at 2R, and ML COP range showed a tendency to increase at this time point. Both AP and ML returned to baseline by 60R. COP velocity was unchanged in AP or ML 2 min following the 32MWT. By 60R, velocity had decreased from baseline in ML and tended to be lower in AP. Data are mean ± SEM; dashed line indicates baseline; * p<0.05 for difference from baseline. AP: anterior-posterior; ML: medial-lateral.

There were main effects of time for COP range in AP (p≤0.04) and a trend in ML (p = 0.09). At 2R, COP range (Fig 2, left) increased during quiet stance in AP (p<0.01) and tended to increase in ML (p = 0.09). Changes in AP and ML COP range returned to baseline by 60R (p≥0.10). There were also main effects of time for COP velocity in both AP and ML (p≤0.01). Post-hoc testing indicated that COP velocity (Fig 2, right) was unchanged from baseline at 2R in AP or ML (p≥0.16), but COP velocity decreased to below baseline at 60R in ML (p = 0.03) and tended to decrease below baseline in AP (p = 0.07). Mean chair rise times did not differ from baseline at 2R or 60R (p≥0.45, Table 2).

### Relationships among high-velocity muscle power, postural control, physical function

**Fig 3:**
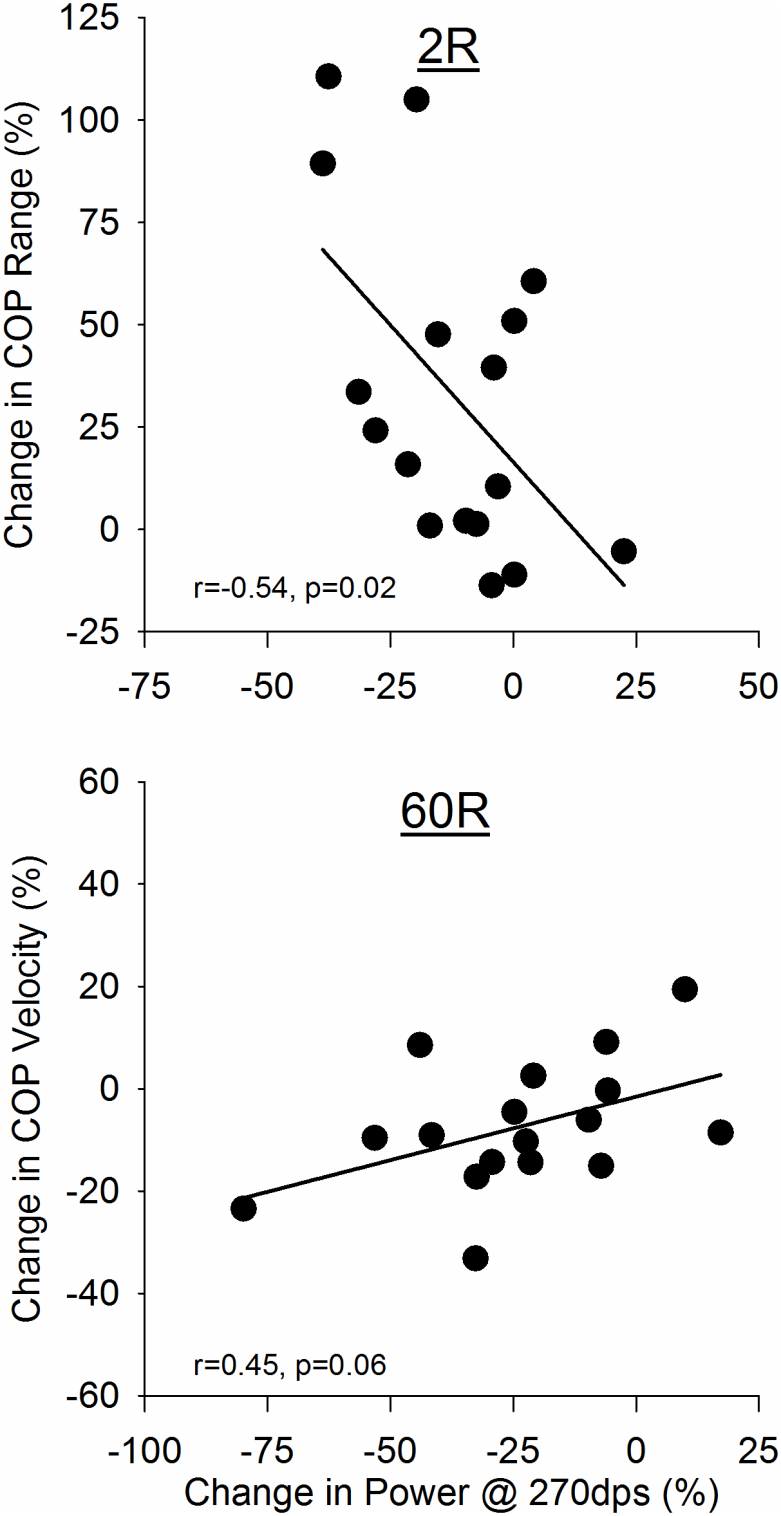
Associations between changes in high-velocity power and postural control. The change in high-velocity (270°·s^-1^) power in response to the 32MWT at 2R was negatively associated with the change in AP COP range (*top*), indicating that fatigue was associated with instability. At 60R, the power deficit at 270°·s^-1^ was positively correlated with COP Velocity (bottom), suggesting that greater residual weakness was associated with increased stability.

**Fig 4:**
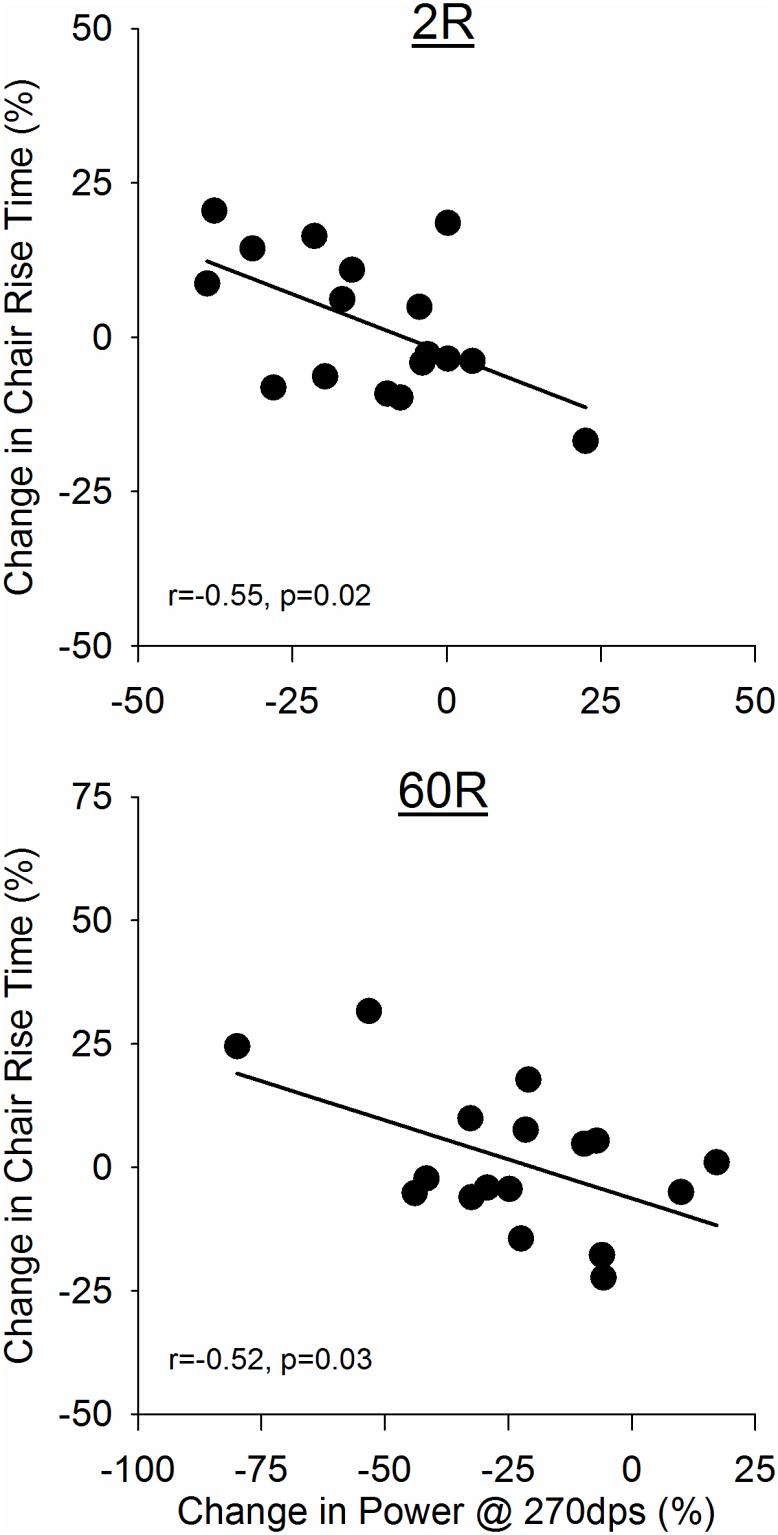
Associations between changes in high-velocity power and chair rise time. Correlations of the percent changes of power at 270°·s^-1^ and chair rise at 2R (middle) and 60R (bottom) showed a significant relationship such that individuals with a greater decrease in power had a greater slowing of chair rise time in response to the 32MWT.

Walking-induced changes in high-velocity power (270°·s^-1^) were associated with changes in postural control and chair rise performance. At 2R (Fig 3, top), changes in power at 270°·s^-1^ were inversely associated with changes in AP COP range; this association was no longer evident by 60R (r = 0.39, p = 0.13, not shown). In contrast, there was no association between changes in power and changes in AP COP velocity at 2R (r = 0.08, p = 0.76, not shown), but by 60R there was a trend for a direct association between these two variables (Fig 3, bottom). At both 2R and 60R, changes from baseline in high-velocity power were inversely associated with changes in chair rise time (Fig 4). No other significant relationships were observed for the changes in power and physical function measures in response to the 32MWT.

## Discussion

The present study provides evidence that, in healthy older women, 32 min of walking at a moderate intensity can negatively affect muscle power production and postural control for at least 60 min following the termination of exercise. While 3 brief “challenges” were inserted into the protocol, the pace used was based on self-selected walking speed during a 400 m overground walk. In support of Hypothesis 1, a moderate-paced walking task caused significant fatigue in the knee extensor muscles of older women who had little evidence of mobility impairments. Following 60 min of recovery, muscle power remained depressed at all velocities, and the magnitude of this power deficit increased with contraction velocity (Hypothesis 2). Hypothesis 3 was partially supported: postural control during quiet stance was altered immediately following the walking task and did not return to baseline within 60 min. In contrast, there was no change in mean chair rise time. Likewise, partial support for Hypothesis 4 was found in the significant associations between changes in muscle power at 270°·s^-1^ and changes in both postural control in the AP direction and chair rise time 2 and 60 min following the walk. Collectively, these results provide evidence of striking alterations in neuromuscular function and postural control in older women following a walking task designed to reflect potential physical challenges encountered in everyday life. Both acute changes associated with muscle fatigue in response to the walk and prolonged alterations that could be expected to impact function in daily activities were observed.

### Fatigue and recovery of muscle power in healthy older women

The 32MWT induced significant knee extensor muscle fatigue in this group of healthy older women. Speed of the 32MWT in the present study (1.35 m·s^-1^) was comparable to the preferred walk speed reported by a population of habitual walkers (1.44 m·s^-1^, [6]). Two minutes following the 32MWT, knee extensor muscle strength deficits ranged from 8% for the MVIC to 13% during the maximal contraction at 270°·s^-1^ (Fig 1). Our observed power deficit was less than the deficit (~30–75%) observed immediately following single-leg, fatiguing knee extensor protocols performed on dynamometers using similar velocities [5]; however, this earlier study reported the force deficit during the final contractions of the fatigue protocol. In the present study, when the two minutes of recovery are taken into account, our strength measures had returned to a level (15% deficit) comparable to that observed after 2 min of recovery following a maximal-contraction knee extensor protocol at 270°·s^-1^ in young and older women (Fig 1, [34]). While the exact amount of fatigue that may have been observed had we been able to measure fatigue immediately at the end of the walk is not known, given the typical rapid initial phase of strength recovery following fatigue, it appears likely that the loss of muscle power immediately following the 32MWT was likely much greater than we observed at 2R. This interpretation would suggest a short-term period (<2 min) of even greater vulnerability in this cohort following the walking exercise.

While prior studies have shown an incomplete recovery of isometric force [13], a key result of this study was that dynamic muscle power did not return to baseline after 60 min of recovery. Furthermore, this power deficit was greater at higher velocities, suggesting the mechanism for loss of high-velocity power following walking may be based more on the failure of the muscle to generate velocity than a failure to produce force. This concept would be consistent with our prior work showing both greater deficits in the torque-muscle size [5] and power-velocity relationships at high velocities [32] and greater fatigue at high velocities [5] in the knee extensor muscles of older compared with younger adults. Further, Power et al [14] reported incomplete recovery of high-speed power following fatiguing dynamometer exercise that coincided with the presence of low-frequency fatigue, a result that implicates impaired excitation-contraction coupling in the decreased ability to produce power. Excitation-contraction coupling failure has been observed in older adults, and has been implicated in the incomplete recovery of isometric force [13]. Further, it is know that low-frequency fatigue can last for at least 24 hours following exercise [15]. Thus, if a similar mechanism contributes to the loss of power observed in the present study, complete recovery of neuromuscular function could be delayed by hours. Other factors, such as increased antagonist coactivation with fatigue, also may have influenced the loss of muscle power reported here. Antagonist (i.e., hamstring) coactivation would reduce the power of contraction by providing resistance against which the knee extensors would have to overcome, thus reducing net power production. While not evaluated in this study, there is evidence to suggest that antagonist coactivation is greater as age, muscle fatigue, and contraction velocity increase [35, 36]. To our knowledge, no studies have followed antagonist coactivation for long durations after fatigue, so the contribution to the power losses at 60R are unclear. Given our results regarding the long-term effects of fatigue induced by walking, clarification of the mechanisms at work here is needed.

It is important to emphasize that the results reported here were obtained in reasonably healthy, community-dwelling older women of modest physical activity levels. Despite the range in ages of the older women, none of the power, postural control, or physical function measures were correlated with age (data not shown). By design, none of the participants in this study had significant mobility impairments at baseline, as indicated by their high SPPB scores. None of these women had a history of falls. Self-reported fatigue, as assessed with the PROMIS questionnaire [25], was on average with the U.S. population. While their 400 m walk time, foot tap speed, activity level, and maximal isometric torque were lower than those of younger individuals reported elsewhere, these values are similar to those observed in other well-functioning older adults [37, 38]. Despite their general good health and function, the older women in this study developed fatigue and postural disturbances that could put them at risk of mobility challenges in response to everyday activities. It is reasonable to expect that the effect of the 32MWT, and similar daily activities, may be greater in persons with existing mobility impairments, sarcopenia or muscle weakness.

### Fatigue and recovery of postural control and physical function

The postural control data suggested immediate effects of the 32MWT on COP range (Fig 2, left), and effects on COP velocity that emerged at 60R (Fig 2, right). Two minutes following the walking task, COP range was increased in the AP direction and tended to be increased in the ML direction, indicating greater sway in the older women during quiet stance. This increase in range was not accompanied by an increase in COP velocity 2 min after the 32MWT. Our COP range results are in agreement with the work of others who have noted increases in COP displacement in older adults following fatiguing calf contractions [19]. In young adults, Nardone et al [18] noted an increase in COP sway that recovered to baseline 15 min following treadmill walking. To our knowledge, the longer-term recovery of postural control following fatigue in older adults has not been evaluated prior to the present study.

Following 60 min of recovery, AP and ML COP range returned to baseline (Fig 2, left). In contrast, decreased ML COP velocity and a trend for decreased AP COP velocity were observed at this time point (Fig 2, right). In both directions, there was a main effect of time for COP velocity. In older adults, increased COP velocity is thought to indicate decreased postural stability by reflecting large postural corrections to maintain upright balance [39]. Here, decreased COP velocity could thus signal that our participants were able maintain postural control. Alternatively, decreased COP velocity in our case might be due to an inability to produce rapid changes in COP. Overall, it is clear that the 32MWT induced short- and long-term alterations in the postural control strategies adopted by healthy older women following a moderate-intensity walking task. Elucidation of the causes and consequences of these alterations warrants further investigation.

The lack of an overall effect of the 32MTW on chair rise time suggests that either the study participants had recovered sufficient physiological reserve 2 and 60 min following the walk to produce the necessary power to perform this task, or that they employed alternative strategies to accomplish this movement. Hortobagyi et al [11] showed that older adults may use up to 80% of their leg strength to rise from a chair, leaving a functional reserve of only 20%. Thus, if fatigue in some of our participants exceeded their functional reserve, they would not have been able to complete the chair rise without an altered motor control or biomechanical strategy. Since power was significantly decreased throughout the hour following the 32MWT, it seems reasonable to surmise that some individuals in our cohort did, in fact, make such alterations. It is also possible that at least some of our participants had a sufficient functional reserve to accomplish the chair-rise task. If this was the case, then in agreement with the non-linear nature of the functional (or physiological) reserve described by Buchner et al [10], large changes in power (i.e., due to fatigue) would result in only small changes in physical function in this subset of the group.

### Relationships among high-velocity muscle power, postural control and physical function

Greater fatigue of high-velocity contractions was associated with greater AP COP range at 2R and slower AP velocity at 60R (Fig 3); these associations were not found with fatigue of isometric or low-velocity contractions. These relationships suggest that reduced power was related to different strategies used to maintain stability at 2R and 60R; the precise mechanism involved in these strategies remains to be determined. Weakness may play a prominent role in instability shortly after the end of exercise, but compensation in balance by 60R may have occurred through improvements in another mechanism, such as somatosensory or vestibular function. Joint proprioception [40] and vestibular input [41] have been shown to be reduced in young adults following exercise. To our knowledge, there are no studies of recovery of these measures following exercise in older adults. It is also possible that, following 60 min of sitting with only intermittent movement after the walking protocol, tissue stiffness was increased, possibly due to antagonist coactivation, edema, small amounts of muscle damage during the exercise, or altered coordination strategies. Increased stiffness about the knee could hinder velocity-dependent knee extensor power production [42], yet provide increased overall ankle stability during quiet stance [43].

The relationships we observed between exercise-induced changes in power and postural control variables were found in the AP, but not ML, direction. Motion in the AP direction has been hypothesized be more dependent on muscle strength than motion in ML, due to the multiple joints that require muscular support in AP [44]. In particular, AP postural control variables were associated with high-velocity power. This result is consistent with Loram and Lakie’s hypothesis [20] that rapid, low-force ballistic contractions are needed to maintain postural control in the AP. While their results are specific to ankle dorsi- and plantar flexor muscle function, which we did not measure here, it is possible that our 32MWT affected these muscles to a degree similar to what we observed for the knee extensors. Thus, greater instability post-fatigue may be due to an inability of the neuromuscular system to make these rapid postural adjustments.

The changes observed here in COP velocity following the walk were modest with respect to the changes in knee extensor power. It seems likely, then, that power was not the limiting factor in determining changes in these variables. While knee extensor strength has been correlated with postural sway in older adults, Menz [45] showed that tactile sensitivity was more predictive of sway than baseline strength. Thus, while strength may play a dominant role during some postural control tasks [2], the impact of strength changes may be secondary to that of other factors such as tactile sensitivity [45] in healthy, older adults. Impaired recovery of power may have a greater effect under more challenging postural control conditions, such as with the eyes closed or during reaching tasks, as well as during dynamic balance tests, such as walking. Likewise, it may be that the association between power loss and postural control changes are more robust in persons with baseline deficits in these and other mobility variables.

At both 2R and 60R, there were significant, indirect associations between changes in high-velocity muscle power and changes in chair rise time in response to the 32MWT (Fig 4). Greater declines in high-velocity power were associated with slower chair-rise times. Hortobayagi et al [11] observed a peak knee extension velocity of 138°·s^-1^ in older adults performing a chair rise. While our observations at 270°·s^-1^ may over-estimate any power loss at 138°·s^-1^, this relationship between power and chair rise performance emphasizes the importance of high-velocity power in maintaining physical function in older adults. Interestingly, despite the significant relationship between the change in high-velocity power and change in chair rise time, 6 individuals at 2R and 7 individuals at 60R had improved chair rise scores despite a reduction in power. The causes of this heterogeneous response are not clear at this time; further research will be necessary to clarify this relationship.

## Conclusions

We provide evidence that 32 min of moderate-intensity walking is sufficient to induce significant fatigue of the knee extensor muscles of healthy older women, and limit power production in a velocity-dependent manner for at least one hour. Notably, fatigue of high-velocity power was associated with changes in postural control and functional performance, both at fatigue and at the end of the recovery period. These results indicate that neuromuscular and physical function may be compromised and postural control strategies altered for some time in healthy older adults following a physical challenge as common as walking. Further, the results of this study suggest a critical need to determine the mechanisms, duration and potential consequences of this period of vulnerability. Additional research is needed to determine how to prevent these lingering decrements in power in order to minimize the risk of falls and disability in older adults.

## Supporting information

S1 File Reduced power, postural control, and chair rise data for individual subjects. (PDF)
