# Supplementary material for: Post-fatigue recovery of power, postural control and physical function in older women
Source: PLoS One. 2017 Sep 7;12(9):e0183483. doi: 10.1371/journal.pone.0183483 (PMC5589131; doi:10.1371/journal.pone.0183483)
Supplement: S1 File — (PDF) [file pone.0183483.s001.pdf]

Subject	dps0_Base	dps0_2r	dps0_60r	dpsV75_Ba	dpsV75_2r	dpsV75_6(	dps30_Bas	dps30_2r	dps30_60r
1	143.66	116.94	109.59	80.06	71.90	65.75	82.10	51.49	40.79
2	102.19	97.64	106.59	116.75	112.24	91.64	42.96	44.13	43.09
3	97.23	93.77	90.44	116.21	86.76	92.80	38.24	32.02	34.13
4	86.76	89.80	84.40	67.33	64.85	61.48	37.59	33.05	34.69
5	106.00	93.40	94.60	66.20	57.00	55.40	46.00	36.80	38.70
6	134.91	109.89	116.18	105.74	103.66	100.63	63.09	56.20	54.41
7	117.12	112.52	105.76	65.02	57.00	46.69	39.38	36.79	38.33
8	149.73	151.56	139.83	94.88	89.64	70.70	65.03	69.80	59.88
9	74.31	67.16	72.91	67.42	64.56	58.35	32.85	32.65	31.96
10	99.03	94.18	89.66	67.44	69.38	58.68	42.50	39.09	37.78
11	134.19	119.57	117.19	52.54	47.70	43.84	52.54	47.70	43.84
12	135.39	107.81	128.42	67.70	63.27	47.57	35.96	32.36	33.55
13	98.96	93.78	96.20	111.93	113.07	89.72	47.26	41.34	34.77
14	107.09	90.40	103.00	70.50	52.16	49.11	45.58	34.86	39.99
15	119.22	132.74	132.62	95.33	99.21	101.72	49.56	51.44	56.64
16	114.82	103.34	101.57	66.63	64.57	61.62	42.46	38.33	38.97
17	92.11	71.15	79.39	39.11	30.95	32.11	39.11	30.95	32.11

dps270_Ba	dps270_2r	dps270_60r		AP_COPRa	AP_COPRa	AP_COPRa	ML_COPRa	ML_COPRa	ML_COPRa
116.09	104.93	82.10		33.31	33.99	22.6	6.92	7.1	10.28
176.64	168.86	159.60		15.1	13.03	14.53	6.68	2.97	4.57
150.74	92.26	30.26		14.96	28.32	15.76	6.38	14.82	18.35
94.52	64.77	110.77		23.63	31.56	22.77	6.11	9.78	5.5
150.00	127.00	101.00		26.64	39.32	35.72	8.96	11.53	13.52
224.07	215.18	168.59		24.32	33.93	30.24	12.42	10.08	19.25
64.10	78.53	60.23		15.71	14.86	24.44	4.84	5.28	5.57
152.44	95.07	167.64		15.17	31.95	35.81	6.41	15.67	7.96
127.54	102.41	98.95		24.1	49.41	17.93	6.79	24.58	4.17
114.94	119.74	77.62		28.28	45.42	39.81	11.31	16.43	9.36
183.89	178.18	103.01		34.29	37.87	21.03	9.12	18.05	15.38
123.71	102.74	114.93		26.93	27.17	39.2	12.32	13.14	14.13
184.15	184.57	145.65		21.75	19.34	37.19	9.29	8.28	10.31
162.77	127.86	76.14		13.3	15.41	15.65	3.48	5.83	7.89
194.00	179.46	113.29		19.32	19.57	23.89	11.49	6.97	7.86
162.07	162.38	152.79		29.06	43.85	42.36	14.87	13.06	17.03
26.72	19.23	20.98		26.54	32.94	29.3	10.26	14.87	11.37

AP_COPVe	AP_COPVe	AP_COPVe	ML_COPVe	ML_COPVe	ML_COPVelocity_60R	ChairRise_	ChairRise_	ChairRise_(
130.79	152.91	112.07	56.76	60.05	57.3	6.78	6.16	6.5
115	98.41	108.01	76.22	68.58	72.93	10.12	10.62	10.6
106.51	106.32	81.53	68.34	72.32	71.25	12.5	13.59	15.56
117.42	115.61	107.35	76.93	80.42	74.19	10.21	11.68	10.31
176.47	185.92	117.94	88.81	92.84	84.37	6.68	7.41	7.34
128.87	124.44	123.03	81.69	67.72	71.82	9.97	9.56	9.53
89.91	107.54	98.13	72.82	73.69	72.83	5.9	4.91	4.85
107.02	142.8	127.81	81.65	89.3	85.75	7.31	8.81	6.94
106.59	129.05	95.6	86.97	103.19	80.37	11.84	11.09	10.13
117.89	139.36	97.62	70.87	75.73	65.96	10.69	10.28	10.04
113.36	183.65	123.05	69.51	80.42	71.23	5.87	5.71	5.56
139.86	142.23	118.76	63.95	79.57	63.22	12.16	12.91	12.81
115.78	110.22	118.72	79.39	74.54	77.48	4.91	5.82	5.78
80.49	81.98	72.76	71.3	73.05	69.57	6.58	7.66	8.66
92.35	75.02	83.97	73.95	79.39	70.49	9.53	8.6	9.31
166.82	175.02	166.23	111.85	109.28	97.62	4.34	4.19	3.37
105.54	103.38	90.35	80.65	76.34	70.88	16.12	14.81	17.34

60R
